# Microbial Synthesis of Non-Natural Anthraquinone Glucosides Displaying Superior Antiproliferative Properties

**DOI:** 10.3390/molecules23092171

**Published:** 2018-08-28

**Authors:** Trang Thi Huyen Nguyen, Ramesh Prasad Pandey, Prakash Parajuli, Jang Mi Han, Hye Jin Jung, Yong Il Park, Jae Kyung Sohng

**Affiliations:** 1 Department of Life Science and Biochemical Engineering, Sun Moon University, 70 Sunmoon-ro 221, Tangjeong-myeon, Asan-si, Chungnam 31460, Korea; nguyenhuyentrang0512@gmail.com (T.T.H.N.); ramesh.pandey25@gmail.com (R.P.P.); parajuli1985@gmail.com (P.P.); gkswkdal200@naver.com (J.M.H.); poka96@sunmoon.ac.kr (H.J.J.); 2 Department of BT-Convergent Pharmaceutical Engineering, Sun Moon University, 70 Sunmoon-ro 221, Tangjeong-myeon, Asan-si, Chungnam 31460, Korea; 3 Department of Biotechnology, The Catholic University of Korea, Bucheon, Gyeonggi-do 14662, Korea; yongil382@catholic.ac.kr

**Keywords:** anti-cancer agents, anthraquinones, glycosyltransferase

## Abstract

Anthraquinones, naturally occurring bioactive compounds, have been reported to exhibit various biological activities, including anti-inflammatory, antiviral, antimicrobial, and anticancer effects. In this study, we biotransformed three selected anthraquinones into their novel *O*-glucoside derivatives, expressing a versatile glycosyltransferase (YjiC) from *Bacillus licheniformis* DSM 13 in *Escherichia coli*. Anthraflavic acid, alizarin, and 2-amino-3-hydroxyanthraquinone were exogenously fed to recombinant *E. coli* as substrate for biotransformation. The products anthraflavic acid-*O*-glucoside, alizarin 2-*O*-*β*-d-glucoside, and 2-amino-3-*O*-glucosyl anthraquinone produced in the culture broths were characterized by various chromatographic and spectroscopic analyses. The comparative anti-proliferative assay against various cancer cells (gastric cancer-AGS, uterine cervical cancer-HeLa, and liver cancer-HepG2) were remarkable, since the synthesized glucoside compounds showed more than 60% of cell growth inhibition at concentrations ranging from ~50 μM to 100 μM. Importantly, one of the synthesized glucoside derivatives, alizarin 2-*O*-glucoside inhibited more than 90% of cell growth in all the cancer cell lines tested.

## 1. Introduction

Anthraquinones are naturally occurring phenolic compounds based on the 9,10-anthraquinone skeleton. They are widely available from plants, microbes, fungi, and lichens [1]. Anthraquinones have various biological benefits [2,3]. Anthraquinones of the *Rubiaceae* family exhibit interesting in vivo biological activities such as antimicrobial [4], antifungal [5], hypotensive and analgesic [6], anti-malarial [7], anti-oxidant [8], antileukemic, and mutagenic functions [9]. Several anthraquinones are widely used in the treatment of cancer. They display cytotoxic activities through interaction with DNA, preferentially at guanine/cytosine-rich sites [10]. Emodin was studied as an agent that could reduce the impact of type 2 diabetes [11] and could inhibit human cytomegalovirus development [12]. Aloe emodin has strong stimulant-laxative action [13] and is found in the gel, soap or leaves of *Aloe vera* and the rhizome of rhubarb (*Rheum rhaponticum*). Chrysazin (known as Dantron) is mainly used in palliative care to counteract the constipating effects of opioids [14]. Quinizarin is an inexpensive dye used to color gasoline and heating oil [15]. It is also used as an intermediate for the synthesis of indanthrene- and alizarin-derived dyes. Alizarin has been used as a prominent red dye, principally for dyeing textile fabrics. Anthraquinone glycosides upon hydrolysis yield the aglycone, which is usually a di-, tri- or tetrahydroxyanthraquinone or a derivative of these compounds. While free anthraquinone aglycones exhibit therapeutic activity, glycosides that enhance solubility are accepted as key active components with various pharmacological actions such as antileukemic, antiseptic, anti-cancer, and antitumor activity [16,17].

**Figure 1:**
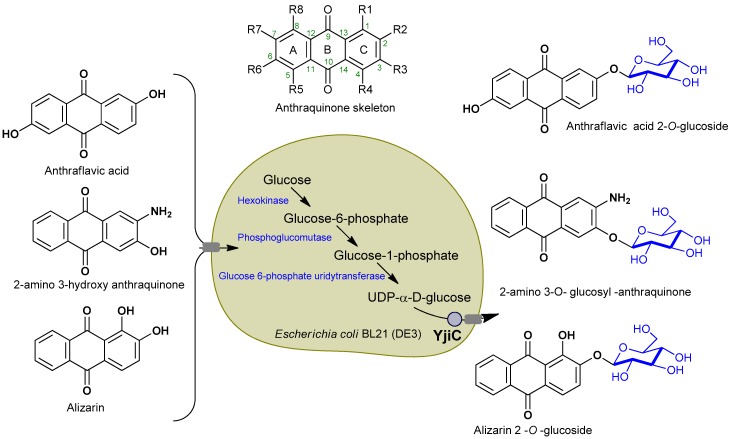
Scheme showing pathway and utilizing *Escherichia coli* indigenous UDP-glucose by *Bacillus* glycosyltransferase for modification of selected anthraquinones into respective glucosides.

Recently, the synthesis of anthraquinone derivatives has attracted significant interest. Various synthesis methods have been reported, the most common of which is the intramolecular condensation of aryl and *O*-arylbenzoic acids, using fuming sulfuric acid, benzoyl chloride, concentrated sulfuric acid, benzoyl chloride, zinc chloride, and POCl_3_/P_2_O_3_C_l4_ as catalyst, which produces anthraquinone derivatives [18]. Anthraquinone and its associated derivatives were previously synthesized by a chemical pathway, although this method was expensive and involved several hurdles [19]. This report is based on the utilization of indigenous *E. coli* sugar (uridine diphosphate (UDP)-glucose) as a sugar donor which will be utilized by the glycosyltransferase to conjugate to different anthraquione derivatives. This process is inexpensive and environmentally friendly. We designed a scheme to consider wild type *E. coli* BL21 (DE3) strain. We then expressed glycosyltransferases from *Bacillus licheniformis* DSM13 (YjiC) and biotransformed three anthraquionones into their respective glucosides (Figure 1). The anticancer activities of all derivatives were assessed and the results were significant compared with those of the corresponding aglycones.

## 2. Results 

### 2.1. Biotransformation of Anthraquinones

The approach was to biosynthesize anthraquinone glucoside utilizing *E. coli* indigenous UDP-glucose as a sugar donor to the expressing glycosyltransefrase (Figure 1). Cultures were prepared for biotransformation reactions with recombinant strain expressing pET28a-YjiC, as explained in the Materials and Methods section. Three anthraquinones: 2-amino-3-hydroxyanthraquinone, anthraflavic acid, and alizarin were chosen for biotransformation. 

Anthraquinones were supplied exogenously to each flask of *E. coli* BL21 (DE3) harboring pET28a-YjiC culture after 20 h of IPTG induction at a final concentration of 0.2 mM. Cultures were allowed to undergo biotransformation for up to 60 h and were then harvested using a double volume of ethyl acetate and analyzed by analytical HPLC-PDA, as described in the Materials and Methods section.

**Figure 2:**
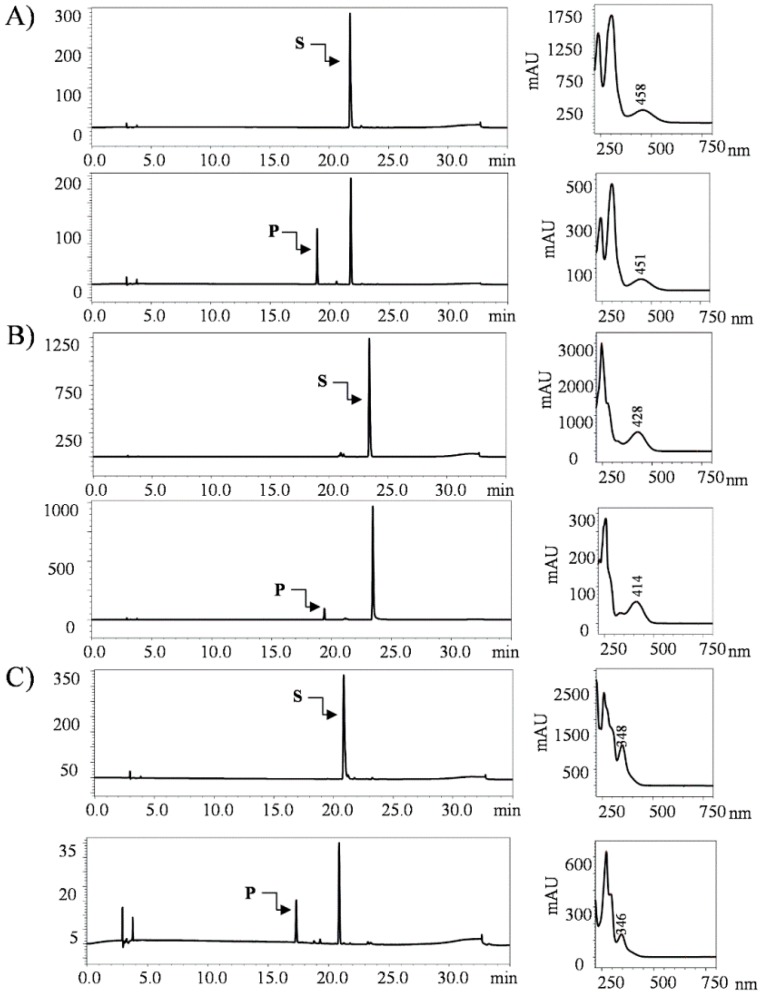
HPLC-PDA chromatogram of biotransformation reaction mixtures compared with respective standards. S refers to the substrate peak and P refers to the product. (**A**) 2-amino-3-hydroxyanthraquinone, (**B**) alizarin, and (**C**) anthraflavic acid.

While analyzing the HPLC-PDA of each sample, product peaks appeared at shorter retention times (*t*_R_) than the substrate peak in each reaction mixture, as expected. New peaks at *t*_R_ ~ 18.9 min for 2-amino-3-hydroxyanthraquinone-*O*-glucoside, of which the substrate peak was at *t*_R_ 21.7 min, *t*_R_ ~ 17.3 min for anthraflavic acid-*O*-glucoside, of which the substrate peak was at *t*_R_ 20.8 min, and *t*_R_ ~ 19.4 min for alizarin-*O*-glucoside, of which the substrate peak was detected at *t*_R_ 23.4 min at the UV absorbance of 420 nm (Figure 2). 

**Figure 3:**
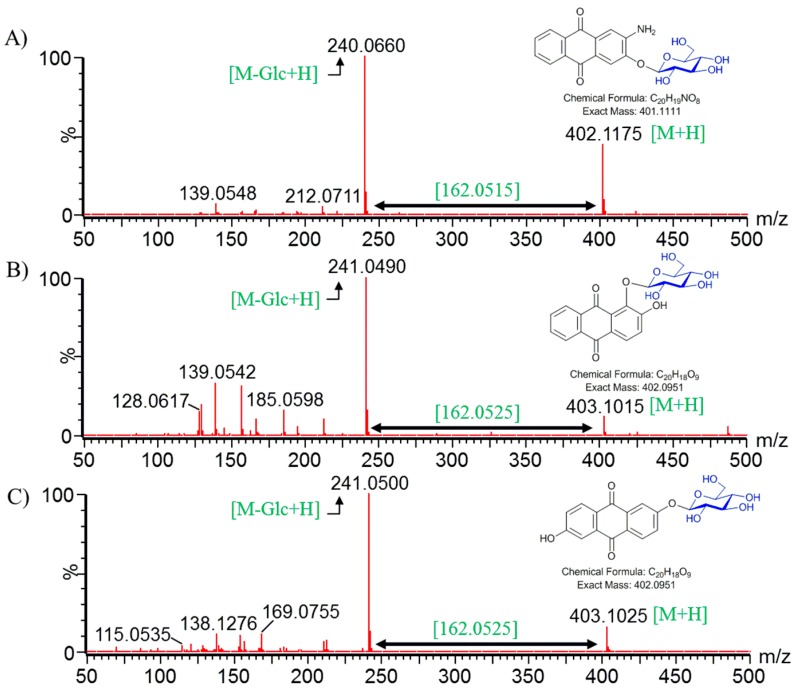
HQ-QTOF ESI/MS analyses of glycosylated (**A**) 2-amino-3-hydroxyanthraquinone; (**B**) Alizarin; and (**C**) anthraflavic acid confirmed by comparing the mass fragments.

These product peaks detected from the glycosylation systems were further analyzed by LC-QTOF-ESI/MS in positive ion mode. The mass spectra displayed an exact mass of 2-amino-3-hydroxyanthraquinone [M + H]^+^ *m*/*z*^+^ ~ 240.0660, while the mass spectrum of [M + H]^+^ *m*/*z*^+^ ~ 402.1175 resembled the glucose conjugated derivative of 2-amino-3-hydroxyanthraquinone. Similarly, anthraflavic acid conjugated to glucose [M + H]^+^ *m*/*z*^+^ ~ 403.1025 and the alizarin conjugated to glucose (which was [M + H]^+^ *m*/*z*^+^ ~ 403.1015) were assumed from the mass analysis as respective mass spectra. The mass spectra were obtained along with their sister fragment of anthraflavic acid [M + H]^+^ *m*/*z*^+^ ~241.0500 and alizarin [M + H]^+^ *m*/*z*^+^ ~241.0490 (Figure 3). In this experiment, while we can detect these substrates and their products, the product level was not easily detected. Therefore, we used another experiment to improve the conversion.

### 2.2. Glucose Supplementation for Production Optimization in Shake Flask

**Figure 4:**
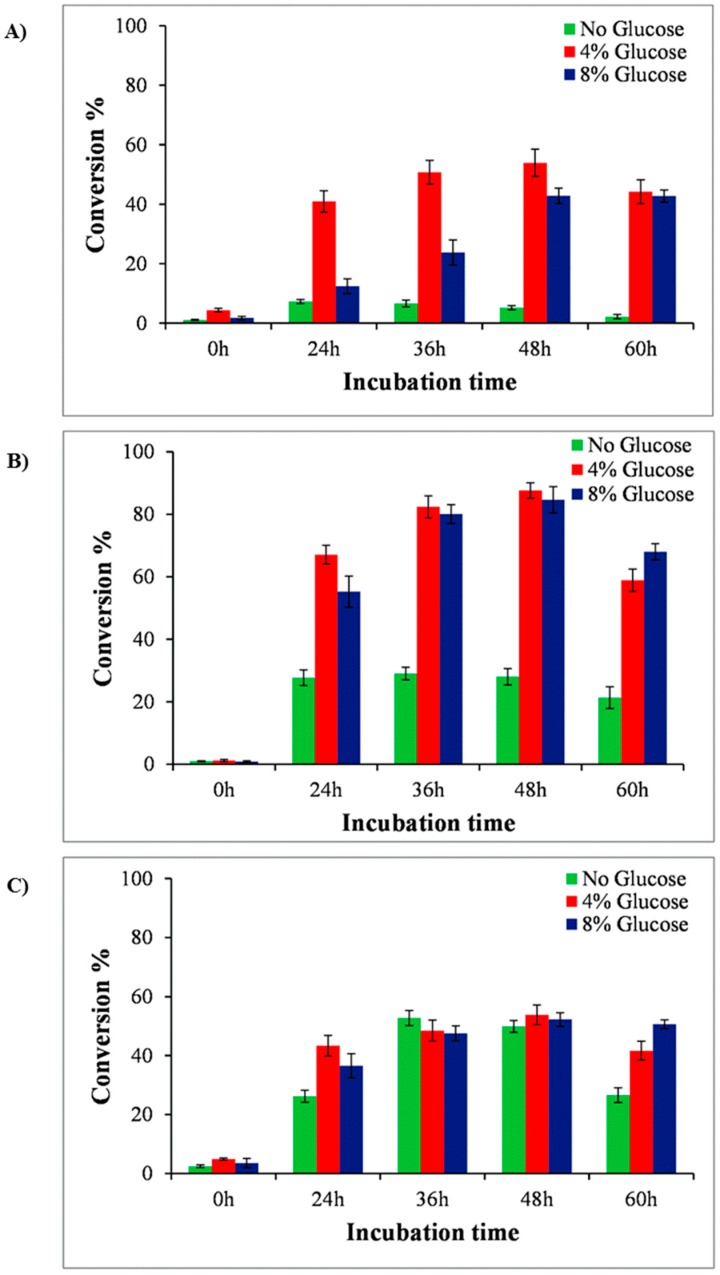
Production profile of anthraquinone-*O*-glucoside at different incubation time intervals and glucose supplementations. (**A**) Alizarin, (**B**) anthraflavic acid, and (**C**) 2-amino-3-hydroxyanthraquinone.

After validating that *E. coli* BL21 (DE3) expressing pET28a-YjiC can biotransform the supplied anthraquinones to their respective glucosides, we considered the optimization of the biotransformation of anthraquinones in shake flasks. Substrates were fed the same amount of 0.2 mM concentration in each flask, while the flasks without the feeding substrates were kept as the control. Samples of 1 mL culture broth from each flask were drawn in each 12 h interval until 60 h. Half of a 0.5 mL sample of culture broth was analyzed for cell density while 0.5 mL of the culture broth was extracted using double volume of ethyl acetate for HPLC sample preparation. While analyzing the production through HPLC, the maximum production of anthraquinone glucoside was achieved from 36 h culture broth (Figure 4).

To enhance the production level of anthraquinone glucosides, we decided to supply extra glucose as carbon source, as reported previously [20] Different concentrations (0%, 4%, and 8%) of glucose were supplemented in separate strain cultures. The production profile of each glucoside changed at different time intervals (from 0 h to 60 h). Previously, while the yields were found to be highest at ~36 h, when these strains were supplemented with glucose, the result showed that production was highest at 48 h (Figure 4). The HPLC-PDA analysis at 48 h resulted in the conversion of approximately 53.89% of alizarin to alizarin-*O*-glucoside in the 4% glucose supplement, while it resulted in the conversion of 5.2% and 42.8% in the 0% and 8% glucose supplements, respectively. Similarly, ~28% and 84.6% anthraflavic acid *O*-glucoside were found in the 0% and 8% glucose supplements in the anthraflavic acid reaction mixture, while 87.5% of the product formation was measured after feeding 4% glucose. The conversion of 2-amino-3-hydroxyanthraquinone to glucoside was limited to 50%, 53.8%, and 52.2% in 0%, 4%, and 8% glucose supplement, respectively (Figure 4 and Figure S1). In each biotransformation reaction, the product formation decreased after 48 h, while the cell growth did not seem to be hampered (data not shown). The decrease in glucoside concentration could be due to the low stability or the deglycosylation properties of YjiC [21].

### 2.3. Comparative Anticancer Activity of Anthraquinone and Their Glucoside Derivatives

**Figure 5:**
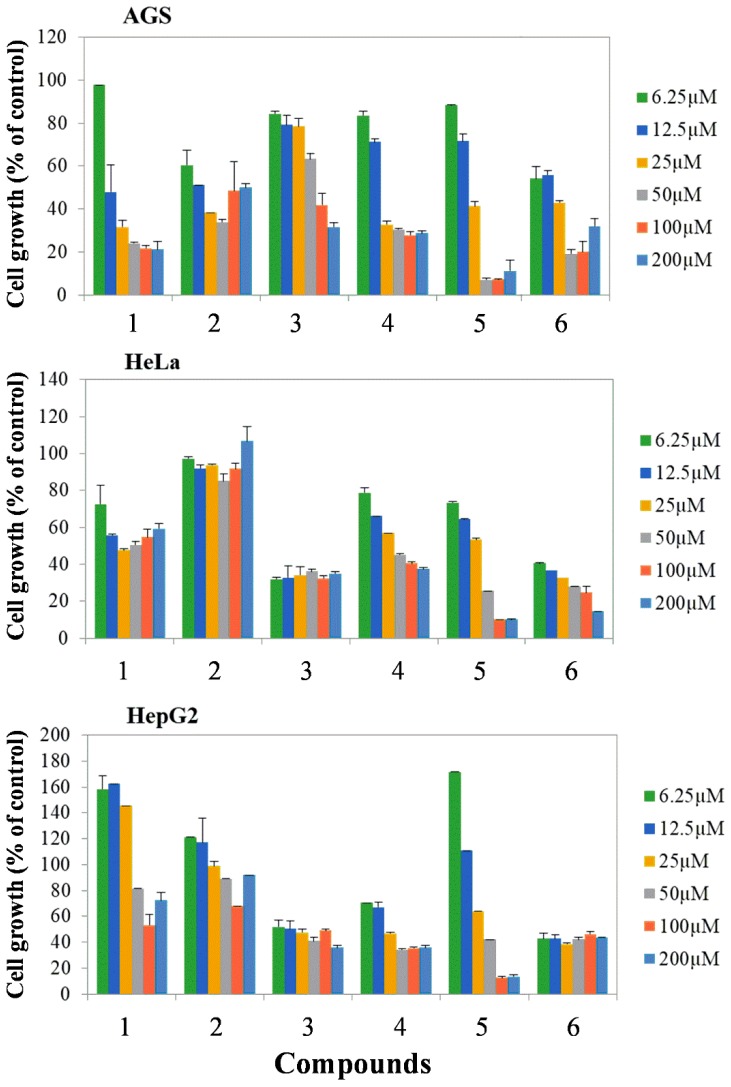
Inhibitory effects of anthraquinones and their derivatives on cancer cell AGS (gastric cancer cell), HeLa (cervical cancer cell), and HepG2 (Liver cancer cell) growth. (1) Anthraflavic acid; (2) Alizarin; (3) 2-amino-3-hydroxyanthraquinone; (4) Anthraflavic acid-*O*-glucoside; (5) Alizarin-*O*-glucoside; (6) 2-amino-3-*O*-glucoxyanthraquinone.

Previous studies have revealed that the anticancer effects of anthraquinones are associated with their suppressive activities of cancer cell proliferation. We thus evaluated the effects of 2-amino-3-hydroxyanthraquinone, anthraflavic acid, alizarin, and their glycosylated derivatives on the proliferation of the AGS, Hela, and HepG2 cell lines. The inhibitory effect of anthraflavic acid-*O*-glucoside on cancer cell growth was comparable to that of anthraflavic acid. Besides, 2-amino-3-hydroxyanthraquinone-*O*-glucoside exhibited greater growth inhibitory effect than 2-amino-3-hydroxyanthraquinone. Furthermore, the inhibitory effects of alizarin-*O*-glucoside were higher than any other anthraquinone glucosides (Figure 5). This result demonstrated the positive inhibitory effect of alizarin-*O*-glucoside on test cancer cell lines compared to other compounds. 

### 2.4. Scale up Production of Alizarin-O-Glucoside in Bioreactor

**Figure 6:**
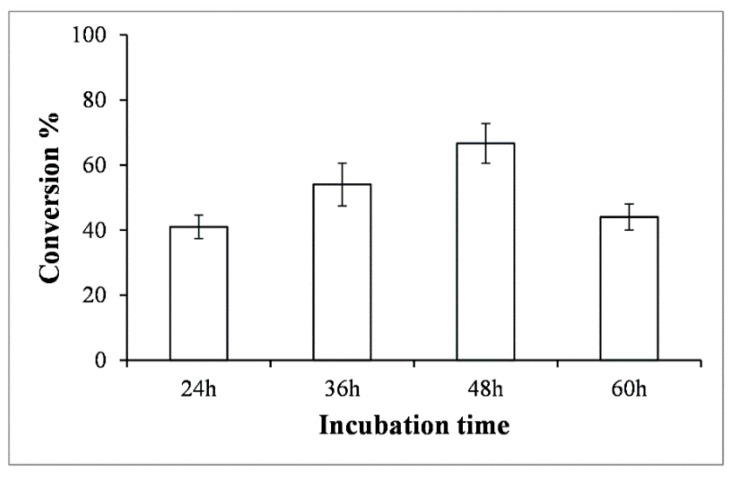
Percentage conversion of alizarin-2-*O*-*β*-d-glucoside in bioreactor at different incubation time intervals.

Based on the anticancer activity of alizarin-*O*-glucoside, we carried out a scale-up experiment for the same molecule to study the feasibility of microbial biosynthesis in a lab scale fermentor. The reaction culture broth was harvested at a regular time interval of 12 h and analyzed by HPLC–PDA to monitor the percentage conversion of alizarin into its glycosides. The HPLC-PDA analysis at 48 h resulted in the conversion of approximately 67% of alizarin to alizarin-*O*-glucoside higher than in the flask (~53.89%). From the 3-L bioreactor, the production of ~265.32 mg of alizarin-*O*-glucoside was achieved (Figure 6).

### 2.5. Structural Elucidation of Potential Anthraquinone Glucoside Derivative 

**Figure 7:**
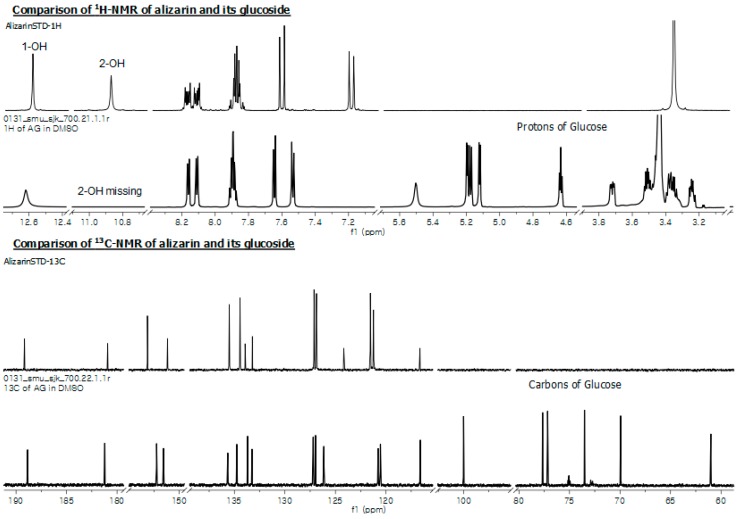
^1^H- and ^13^C-NMR of alizarin and alizarin-2-*O*-*β*-d-glucoside.

The product alizarin-*O*-glucoside was purified using prep-HPLC. The purified fraction was concentrated using a rotary evaporator, then lyophilized to remove water content, and then subjected to various NMR analyses. An anomeric proton was detected at 5.18 ppm with *J* value 7.4 Hz referring the conjugation of glucose at beta (*β*) configuration while anomeric carbon was at 100 ppm (Figure 7, Figures S4 and S5, and Table 1). Evidence was further gathered from the proton NMR analysis of the glucoside product of alizarin, by a missing singlet peak for the hydroxyl group in 2-OH, while the similar peak for 1-OH is intact at 12.62 ppm (Figures S2 and S4). This indicates that the possible sugar conjugation is at 2-OH of alizarin. This result was further supported the correlation between the observed anomeric carbon and anomeric proton revealed by HSQC (Figure S6). The carbon number 2 (C-2) of the alizarin signal appearing at *δ* 151.57 ppm has a direct relation with the observed anomeric proton at *δ* 5.18 ppm in HMBC (Figure S7). This confirms that the glycosylation position was at 2-OH of alizarin, and thus the product is alizarin 2-*O*-*β*-d-glucoside. Furthermore, the spectra for glucose moieties were present at respective places in both ^1^H- (3.0–5.5 ppm) and ^13^C-NMR (60–100 ppm) analyses. The NMR analyses were compared with the previously published report [22] and spectral database for organic compounds SDBS (http://sdbs.db.aist.go.jp).

## 3. Discussion

Anthraquinones are the largest group of natural pigments, with potential applications in various fields, such as an anticancer, antibacterial agents, and anti-inflammatory agents in pharmaceutical medicine [2,23,24]. The industrial significance of anthraquinones is as synthetic dyes providing brilliant colors, providing the natural red chromogen [25], and in the application of the production of hydrogen peroxide [26], etc. These phenolic compounds can be naturally obtained from various sources such as plant, bacteria, fungi, and lichens [27,28]. These compounds are attracting more attention due to the various key factors as aforementioned. While type II polyketide synthase is responsible for anthraquinone biosynthesis in fungus, reports regarding its biosynthesis and modification in microbial hosts are quite limited [29]. The microbial production of such valuable compounds is performed using simple biotransformation techniques, or is used to enhance production using genetic engineering, the synthetic biological tool is an affirmative practice and certainly increases yields [30]. We considered a versatile post-modifying enzyme, the glycosyltransferase (YjiC), that has revealed potential in glycosylating various classes of natural products including anthraquinone itself [31,32,33]. A zero engineered wild type *E. coli* strain was considered to express YjiC for whole-cell biotransformation. The YjiC could catalyze glycosylation reaction over different anthraquinones that were fed exogenously and were confirmed by HPLC-PDA and HQ-QTOF ESI/MS (Figure 2 and Figure 3). Among the products, alizarin-*O*-glucoside, 2-amino-3-*O*-glucoxy anthraquinone, and anthraflavic acid-*O*-glucoside were confirmed by mass analysis (Figure 3) while alizarin-*O*-glucoside was elucidated using various NMR analyses. 

For production enhancement, glucose was used as a supplemental carbon source. Different concentrations of glucose affected the yield and cell density. As can be seen in Figure 4, while a high concentration of glucose (8%) improved the production level, maximum cell growth was achieved within 24 h (growth curve not shown). However, 4% glucose supplement was optimized and favored enhancement in production by maintaining the cell growth and production yield within 48 h compared to the previous experiment (Figure 2). 

Microbial production of emodin *O*-glucoside, aloe-emodin *O*-glucoside using YjiC [33], chrysophanol 8-*O*-*β*-d-glucoside, physcion 8-*O*-*β*-d-glucoside, emodin 6-*O*-*β*-d-glucoside, and aloe-emodin 1-*O*-*β*-d-glucoside were produced by screening 21 different fungal strains for respective anthraquinones biotransformation reported previously [34]. However, all of their biological activities remain unclear, although emodin and aloe-emodin glucoside have shown anti-proliferative activity against some cancer cell lines [33]. Since anthraquinones are potential anticancer agents [35], we synthesized three different *O*-glucosides of 2-amino-3-hydroxyanthraquinone, anthraflavic acid, and alizarin in recombinant *E. coli* strains. These compounds were investigated for anticancer activity against gastric cancer-AGS, uterine cervical cancer-HeLa and liver cancer-HepG2 cells (Figure 5). During the anti-proliferative assay using AGS, HeLa, and HepG2 cancer cell lines, the anthraflavic acid *O*-glucoside and 2-amino-3-*O*-glucosyl anthraquinone revealed more enhanced inhibitory activity than its aglycones, while alizarin *O*-glucoside was more effective than alizarin and other compounds (Figure 5). Although this is a preliminary study for anti-cancer assay, this result suggests that this compound could be developed as an anticancer lead agent in the future. At this point, we needed to determine the exact chemical structure of alizarin *O*-glucoside. Thus, the prep-HPLC purified compound was analyzed with various NMR spectroscopies to elucidate the chemical structure to be alizarin-2-*O*-*β*-d-glucoside (Figure 7). Since other molecules exhibited less activity than alizarin-2-*O*-*β*-d-glucoside, we did not require further structural elucidation of these compounds.

This study provides a comprehensive view of the modification of anthraquinones via biotransformation approach, expressing glycosyltransferase in *E. coli* strain. Anticancer activities of these compounds were assayed against three different cancer cell lines. Among the synthesized derivatives of anthraquinones, alizarin-2-*O*-*β*-d-glucoside was the most effective comparatively in low concentration. This compound could be further investigated in vivo to validate its further potency.

## 4. Materials and Methods

### 4.1. General Procedures

Anthraquinones (2-amino-3-hydroxyanthraquinone, anthraflavic acid, and alizarin) were purchased from Tokyo Chemical Industry (Tokyo, Japan). All other chemicals and reagents were of highest chemical grade. *E. coli* BL21 (DE3, Stratagene, La Jolla, CA, USA) was used as expression and biotransformation hosts. Restriction enzymes were used either from Takara Bio. Inc. (Kusatsu, Japan) or Promega (Madison, WI, USA). Luria-Bertani (LB) plates and broth media supplemented with an appropriate antibiotic (kanamycin 50 μg/mL) was used for the *E. coli* growth, colony selection, culture preparation, and biotransformation. Fermentation was conducted in LB medium to enhance production and collection for biological activity. 

### 4.2. Vectors and Recombinant Strains

The previously constructed recombinant vector pET28a-YjiC [36] was used for recombinant strain construction. The vector was confirmed by restriction endonuclease activity digestion with *Bam*HI/*Xho*I, followed by transformation into wild type *E. coli* BL21 (DE3) for biotransformation study.

### 4.3. Culture Preparation and Whole Cell Biotransformation

Seed culture of *E. coli* strains harboring pET28a-YjiC was prepared in 5 mL LB medium supplemented with 50 μg/mL kanamycin for the maintenance of recombinants in each case, and was then incubated overnight at 37 °C. Approximately 500 μL of seeds were transferred to the same medium (50 mL) and incubated at 37 °C until the cells’ optical density reached 600 nm (OD_600nm_) reached 0.5–0.7, 0.5 mM isopropyl *β*-d-1-thiogalactopyranoside (IPTG) was added to induce protein expression, followed by incubation for 18 h at 20 °C. Since substrates such as 2-amino-3-hydroxyanthraquinone, anthraflavic acid, and alizarin are dissolved in DMSO, we prepared 50 mM stock of each substrate for exogenous supply to the 20 h incubated induced culture prepared. Each substrate at a concentration of 0.2 mM was fed and was allowed to biotransform into the respective products. At the same time, in each case, recombinant strains expressing only vector pET28a(+) without the gene were also fed by the same concentration of anthraquinones as that for the control experiment. After 60 h of incubation, all biotransformation cultures (including controls) were harvested by transforming into a separating funnel followed by the addition of double volume of ethyl acetate and vertical shaking for 30 min then the aqueous and organic layers of the cultures were allowed to settle for another 30 min. The organic ethyl acetate layer in each case was transferred and evaporated using a rotatory evaporator. The final remaining sample was dissolved in 1 mL methanol. This sample was directly analyzed using a high performance liquid chromatogram connected to a photo diode array (HPLC-PDA) and high-resolution quadruple time-of-flight electrospray ionization-mass spectrometry (HQ-QTOF ESI/MS) analysis. 

### 4.4. Whole Cell Biocatalysis in Bioreactor

The recombinant strain of *E. coli* BL21 (DE3) harboring pET28a-YjiC was cultured and the seed was prepared for fermentation. Fermentation was conducted under aerobic condition in LB medium, supplementing glucose as the carbon source. Most protocols followed were similar to those described in our previous reports [20,37]. For the production of anthraquinones glucoside on a large scale, we used a 5-L fermentor system (Biotron, Incheon, Korea). We prepared 3-L of LB medium for fermentation. The seed culture of the recombinant strain expressing glycosyltransferase was cultured in a shake flask (100 mL medium) as an inoculum for the fermentor. Culture was grown overnight at 37 °C in a shaking incubator. For fermentation, the pH meter and the dissolved oxygen (DO) probe were calibrated according to the manufacturer’s protocol. The pH was maintained at 7.0 through the process, using commercially available ammonium hydroxide, while the DO level was maintained at >95% during the entire fermentation period. When the optical density of the culture at 600 nm reached 10, the culture was induced by lactose and the temperature was lowered to 20 °C. After 6 h of culture induction, 240 mg of anthraquinone including 2-amino-3-hydroxyanthraquinone, anthraflavic acid, and alizarin (approximately 0.3 mM final concentration) was added to the culture medium for biotransformation. The fermentor was continuously run for 60 h, maintaining the DO and pH, as mentioned. In each 12-h interval, 100 mL of 50% glucose (autoclaved) was supplied as the carbon source for proper growth. At the end of fermentation, the culture medium was harvested, adding a double volume of ethyl acetate in a separating funnel while vigorously shaking. The aqueous and organic layers were then left to settle. The organic layer was then evaporated using a rotary evaporator to concentrate the samples. The final samples were dissolved in methanol and subjected to purify the products using preparative-HPLC.

### 4.5. Analytical Procedures

From the samples prepared, 20 μL volume was injected and directly analyzed by HPLC-PDA (Shimadzu, Kyoto, Japan; SPD-M20A Detector) using a reverse phase C_18_ column (Mightysil-RP-18GP, 250 × 4.6mm, Kanto Chemical, Tokyo, Japan). The binary mobile phase was composed of solvent A (HPLC grade water + 0.05% trifluoro acetic acid) and solvent B (100% acetonitrile). The total flow rate was maintained as 1 mL/min for a 35-min program. ACN concentrations were 10% (0–10 min), 20% (10–25 min), 100% (25–28 min), 70% (28–30 min), and 10% (30–35 min).

Products were purified by prep-HPLC with a C_18_ column (YMC-Pack ADS-AQ (250 × 20 mm I.D., 10 µm) connected to a UV detector set at 420 nm using a 35 min binary program with ACN concentrations. The ACN concentrations were as follows: 10% (0–10 min), 20% (10–15 min), 50% (15–20 min), 70% (20–25 min), 90% (25–30 min), 50% (30–34 min), and 20% (34–35 min). Purified products were then completely dried in a lyophilizer and used for structural elucidation and bioactivity.

High resolution quadruple time-of-flight electrospray ionization-mass spectrometry (HQ-QTOF ESI/MS) spectra were obtained on ACQUITY (UPLC; Waters, Milford, MA, USA) coupled with SYNAPT G2-S (Waters, Milford, MA, USA). For structural elucidation of biotransformed metabolites, samples including the standards were dissolved in dimethyl-sulfoxide-*d*_6_ (Sigma-Aldrich, St. Louis, MO, USA). Nuclear magnetic resonance (NMR) was conducted by analyzing ^1^H-, ^13^C-NMR with two 2D NMR spectroscopies (heteronuclear single-quantum correlation [HSQC] and heteronuclear multiple-bond correlation [HMBC]). Standard molecules were analyzed using a 300 MHz Bruker BioSpin NMR instrument (Karlsruhe, Germany). Structures of metabolites were elucidated using MestReNova 11.0 program (Mestrelab Research S.L., Santiago de Compostela, Spain). 

### 4.6. Inhibitory Effects of Derivatives on Cancer Cell Growth

Gastric carcinoma cells (AGS) were maintained at the Roswell Park Memorial Institute (RPMI) 1640 medium containing 10% fetal bovine serum (FBS). Cervical carcinoma (HeLa) and hepatocarcinoma cells (HepG2) were grown in Dulbecco’s modified Eagle’s medium (DMEM) supplemented with 10% FBS. All cells were maintained at 37 °C in a humidified 5% CO_2_ incubator. For cell growth assay, various cancer cells were plated at 2 × 10^3^ cells/well in 96-well culture plates. Compounds were added to each well with various concentrations, and the cells were incubated for 72 h. Cell growth was measured using a 3-(4,5-dimethylthiazol-2-yl)-2,5-diphenyltetrazolium bromide (MTT) colorimetric assay. 50 μL of MTT (2 mg/mL stock solution) was added and the plates were incubated for an additional 4 h. After removal of the medium, 100 μL of DMSO was added. Absorbance was measured at 540 nm using a Multiskan^®^ Spectrum microplate spectrophotometer (Thermo Scientific, Waltham, MA, USA,).

## Figures and Tables

**Table 1:** Comparison of ^1^H- and ^13^C-NMR spectra of alizarin and alizarin 2-*O*-glucoside measured in DMSO-*d_6_*. Multiplicities are indicated by s (singlet), d (doublet), t (triplet), q (quartet), and m (multiplet), including coupling constant *J*.

Position	^1^H-NMR		^13^C-NMR	
	Alizarin	Alizarin 2-*O*-*β*-d-Glucoside	Alizarin	Alizarin 2-*O*-*β*-d-Glucoside
1-OH	12.59 (*s*, 1H)	12.62 (*s*, 1H)	153.15	155.25
2-OH	10.88 (*s*, 1H)	-	151.17	151.56
3	7.20 (*d*, *J* = 8.3Hz, 1H)	7.53 (*d*, *J* = 8.55Hz, 1H)	121.55	120.78
4	7.62 (*d*, *J* = 8.3Hz, 1H)	7.64 (*d*, *J* = 8.45Hz, 1H)	121.23	120.55
5	8.15 (*m*, 1H)	8.14 (*ddd*, *J* = 35.05, 7.19, 1.82 Hz, 1H)	126.88	126.97
6	7.89 (*m*, 1H)	7.89 (*td*, *J* = 6.81, 6.78, 1.72 Hz, 1H)	133.44	134.76
7	7.89 (*m*, 1H)	7.89 (*td*, *J* = 6.81, 6.78, 1.72 Hz, 1H)	135.51	135.65
8	8.15 (*m*, 1H)	8.14 (*ddd*, *J* = 35.05, 7.19, 1.82 Hz, 1H)	127.12	127.20
9	-	-	180.93	181.21
10	-	-	189.16	188.85
11	-	-	133.22	133.26
12	-	-	133.94	133.69
13	-	-	116.64	116.60
14	-	-	124.17	126.14
1′	-	5.17 (*d*, *J* = 7.4 Hz, 1H)	-	100.00
2′	-	3.36 (*ddd*, *J* = 17.8, 15.0, 8.8 Hz, 1H)	-	73.51
3′	-	3.36 (*ddd*, *J* = 17.8, 15.0, 8.8 Hz, 1H)	-	77.17
4′	-	3.49 (*m*, 1H)	-	77.66
5′	-	3.25 (*dd*, *J* = 9.21, 5.23 Hz, 1H)	-	69.94
6′a	-	3.72 (*ddd*, *J* = 11.92, 5.33, 2.13 Hz, 1H)	-	61.01
6′b		3.49 (m, 1H)		

## Supplementary Materials

The following are available online at, Figure S1: Comparison of glucose concentration based on the recombinant strain in 48 h incubation. Maximum conversion of anthraquinone to respective anthraquinone glycosides were achieved while supplementing 4% additional glucose in the medium. (A) Alizarin; (B) Anthraflavic acid; (C) 2-amino 3-hydroxyanthraquinone. S stands for substrate peak while P stands for product, Figure S2: ^1^H-NMR of alizarin, Figure S3: ^13^C-NMR of alizarin, Figure S4: ^1^H-NMR of alizarin-2-*O*-*β*-d-glucoside, Figure S5: ^13^C-NMR of alizarin-2-*O*-*β*-d-glucoside, Figure S6: HSQC correlation of alizarin 2-*O*-*β*-d-glucoside, Figure S7: HMBC correlation of alizarin 2-*O*-*β*-d-glucoside.
