# Supplementary material for: Microbial Synthesis of Non-Natural Anthraquinone Glucosides Displaying Superior Antiproliferative Properties
Source: Molecules. 2018 Aug 28;23(9):2171. doi: 10.3390/molecules23092171 (PMC6225150; doi:10.3390/molecules23092171)

Supplementary Information

Microbial Synthesis of Non-Natural Anthraquinone Glucosides Displaying Superior Antiproliferative Properties

Trang Thi Huyen Nguyen [1,†], Ramesh Prasad Pandey [1,2,†], Prakash Parajuli [1], Jang Mi Han [1], Hye Jin Jung [1,2], Yong Il Park [3] and Jae Kyung Sohng [1,2,*]

[1] Department of Life Science and Biochemical Engineering, Sun Moon University, 70 Sunmoon-ro 221, Tangjeong-myeon, Asan-si, Chungnam 31460, Korea; nguyenhuyentrang0512@gmail.com (T.T.H.N.); ramesh.pandey25@gmail.com (R.P.P.); parajuli1985@gmail.com (P.P.); gkswkdal200@naver.com (J.M.H.); poka96@sunmoon.ac.kr (H.J.J.)

[2] Department of BT-Convergent Pharmaceutical Engineering, Sun Moon University, 70 Sunmoon-ro 221, Tangjeong-myeon, Asan-si, Chungnam 31460, Korea

[3] Department of Biotechnology, The Catholic University of Korea, Bucheon, Gyeonggi-do 14662, Korea; yongil382@catholic.ac.kr

* Correspondence: sohng@sunmoon.ac.kr; Tel: +82-(41)-530-2246; Fax: +82-(41)-530-8229

† These authors contributed equally to this work.

Figure S1. Comparison of glucose concentration based on the recombinant strain in 48 h incubation. Maximum conversion of anthraquinone to respective anthraquinone glycosides were achieved while supplementing 4% additional glucose in the medium. A) Alizarin, B) Anthraflavic acid, C) 2-amino 3- hydroxyanthraquinone. S stands for substrate peak while P stands for product

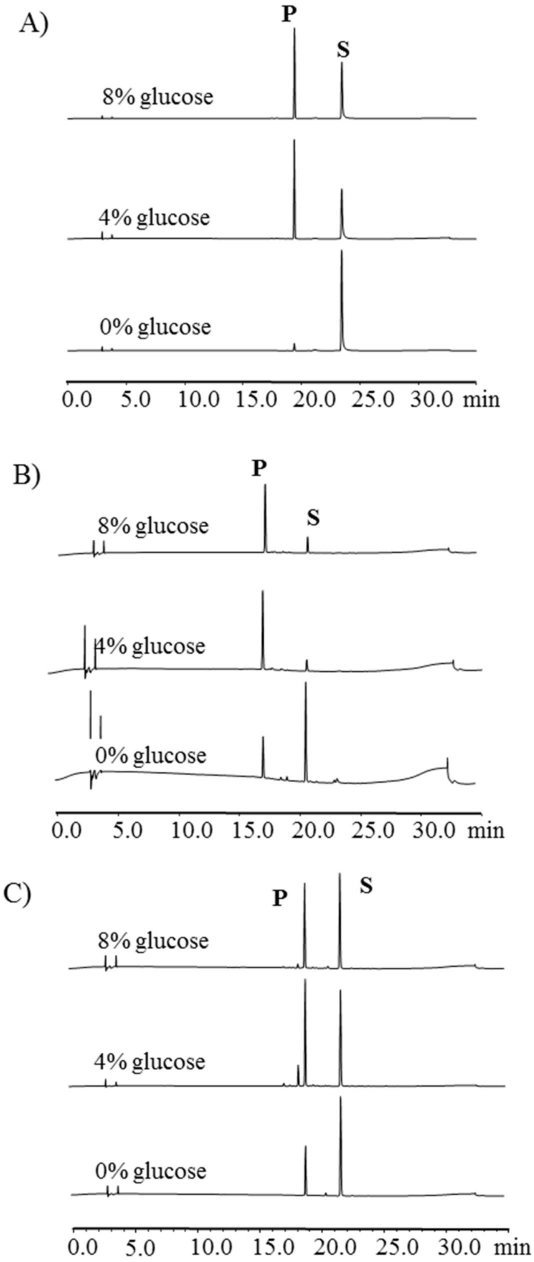

Figure S2. ^{1}H NMR of alizarin

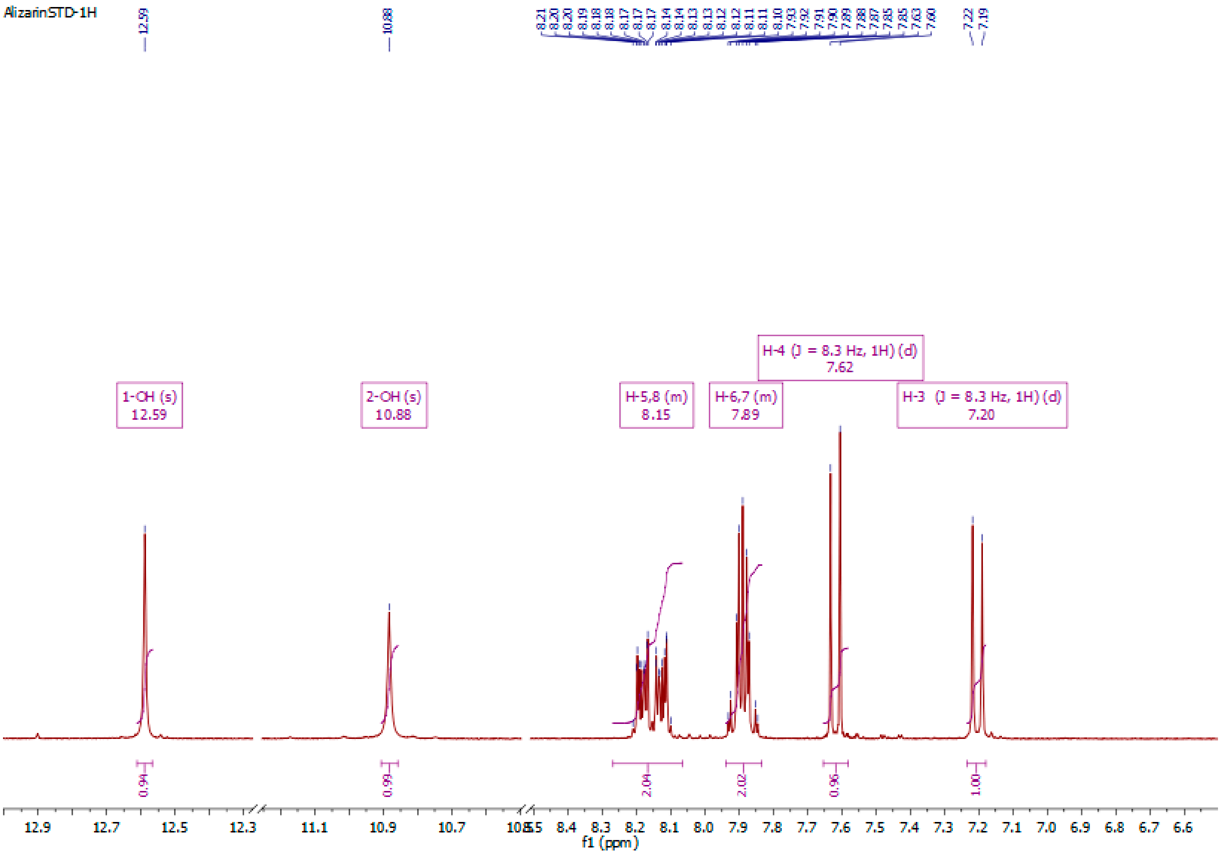

Figure S3. ^{13}C NMR of alizarin

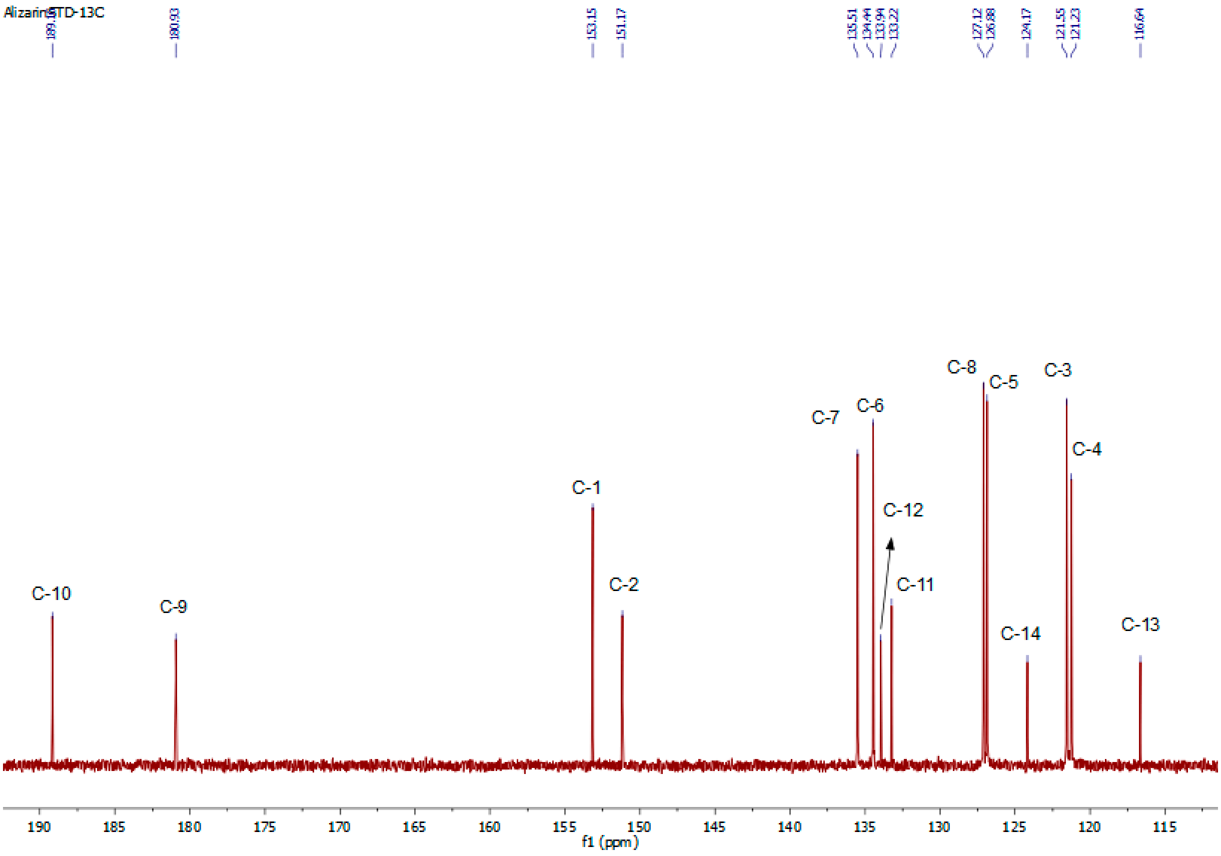

Figure S4. ^{1}H NMR of alizarin-2-*O*-*β*-D-glucoside

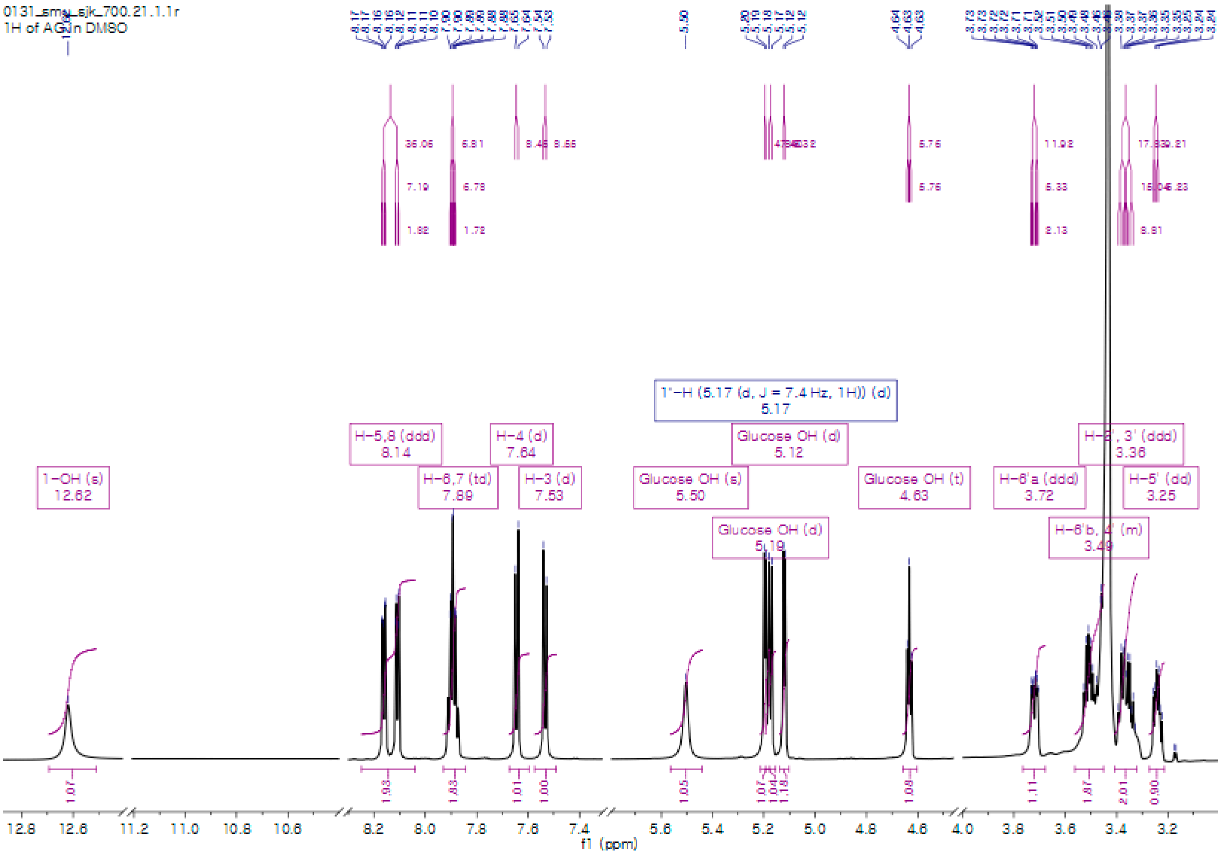

Figure S5. ^{13}C NMR of alizarin-2-*O*-*β*-D-glucoside

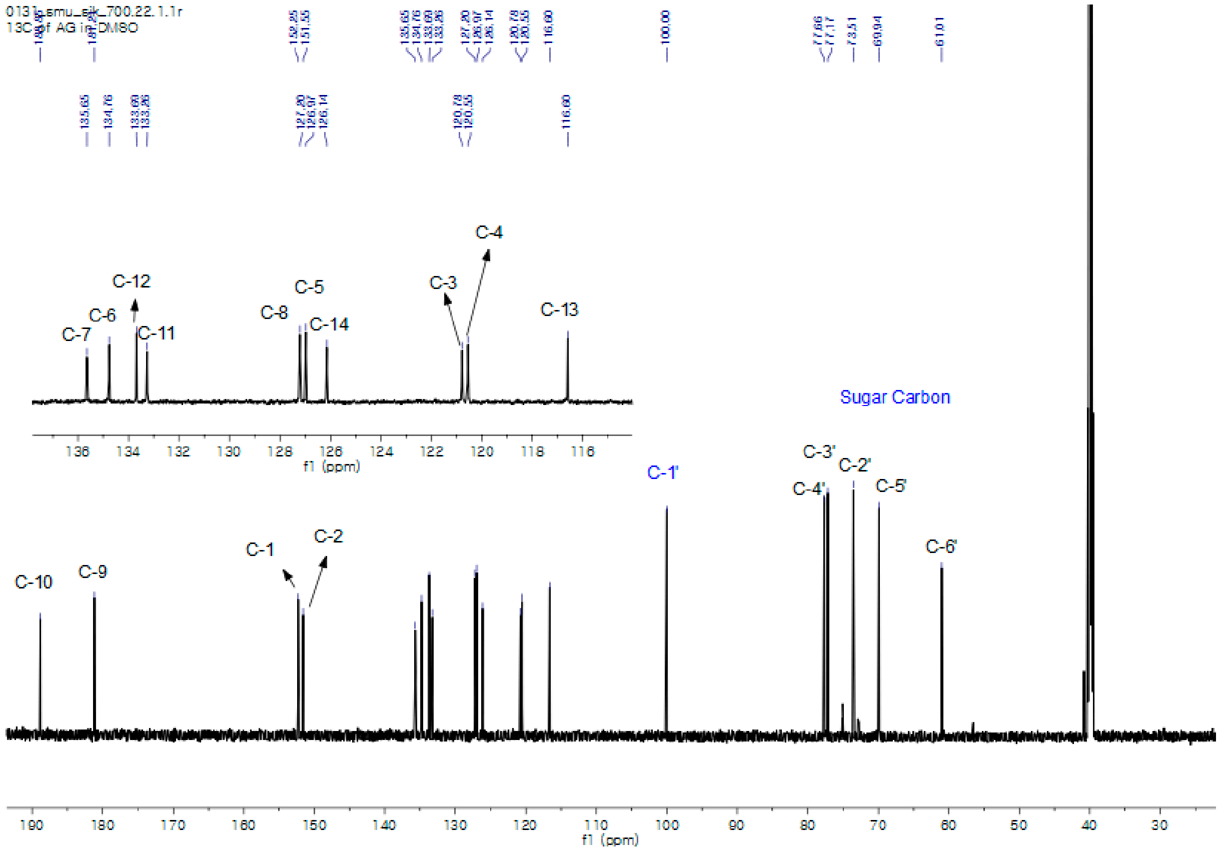

Figure S6. HSQC correlation of alizarin 2-*O*-*β*-D-glucoside

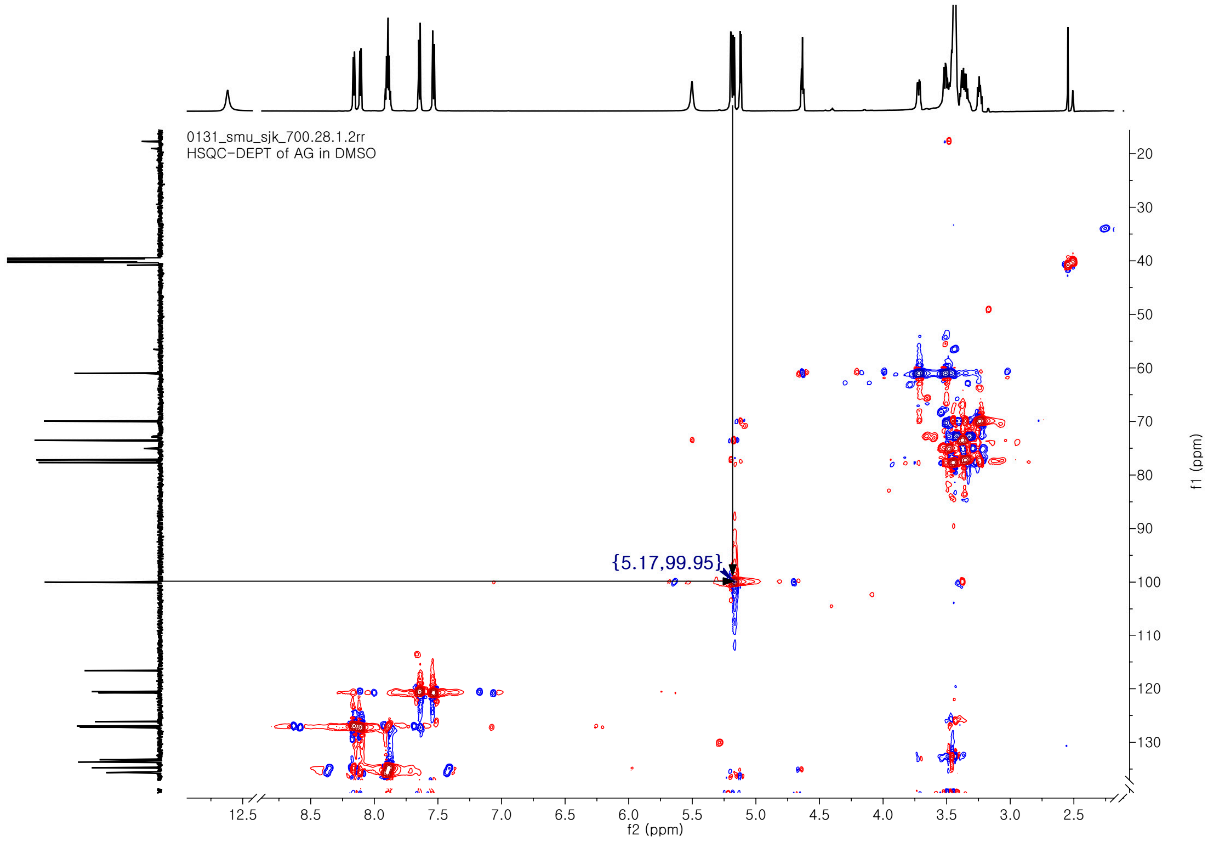

Figure S7. HMBC correlation of alizarin 2-*O*-*β*-D-glucoside

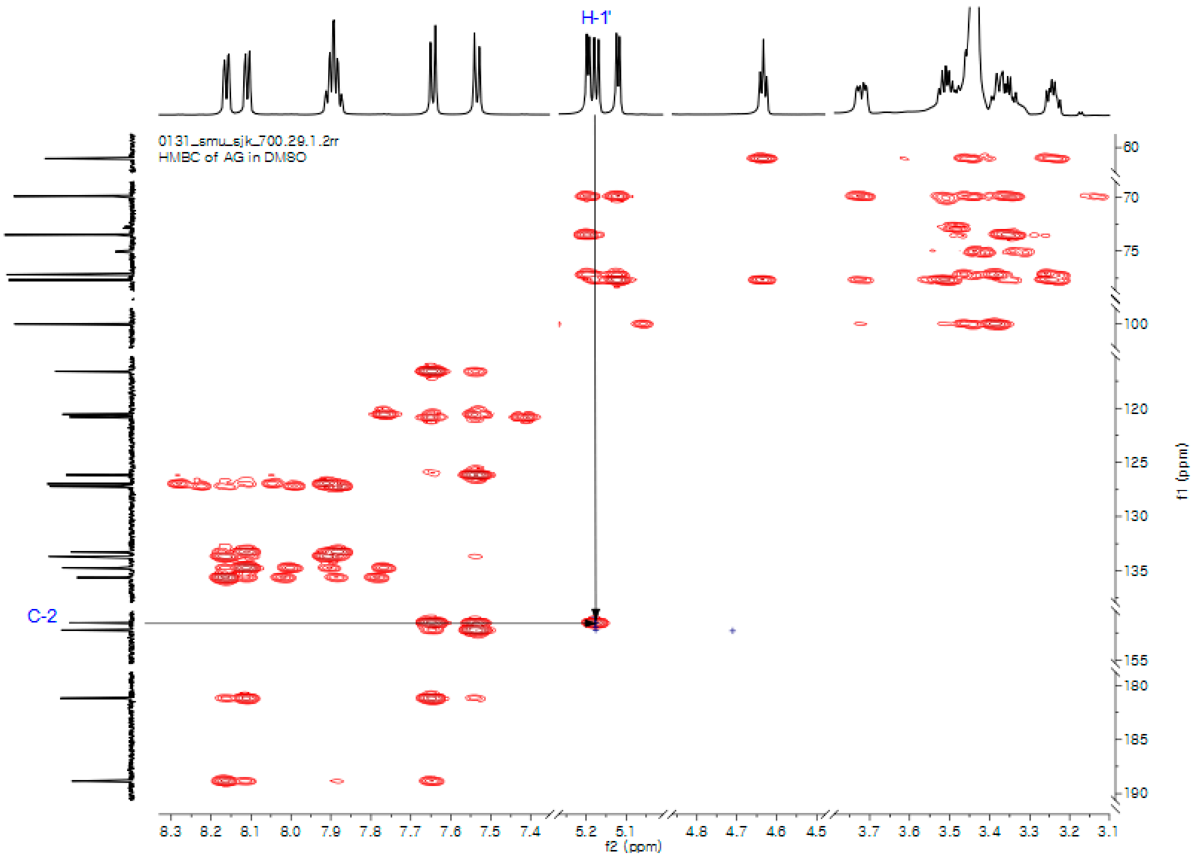

Supplement: Supplementary file 1 [file molecules-23-02171-s001.pdf]
